# Isolation, characterization and heterologous expression of a novel chitosanase from *Janthinobacterium *sp. strain 4239

**DOI:** 10.1186/1475-2859-9-5

**Published:** 2010-01-22

**Authors:** Mads G Johnsen, Ole C Hansen, Peter Stougaard

**Affiliations:** 1 Bioneer A/S, Kogle Allé 2, DK-2970 Hørsholm, Denmark; 2 Section of Genetics and Microbiology, Department of Agriculture and Ecology, Faculty of Life Sciences, University of Copenhagen, Thorvaldsensvej 40, DK-1871 Frederiksberg C, Denmark

## Abstract

**Background:**

Chitosanases (EC 3.2.1.132) hydrolyze the polysaccharide chitosan, which is composed of partially acetylated β-(1,4)-linked glucosamine residues. In nature, chitosanases are produced by a number of Gram-positive and Gram-negative bacteria, as well as by fungi, probably with the primary role of degrading chitosan from fungal and yeast cell walls for carbon metabolism. Chitosanases may also be utilized in eukaryotic cell manipulation for intracellular delivery of molecules formulated with chitosan as well as for transformation of filamentous fungi by temporal modification of the cell wall structures.

However, the chitosanases used so far in transformation and transfection experiments show optimal activity at high temperature, which is incompatible with most transfection and transformation protocols. Thus, there is a need for chitosanases, which display activity at lower temperatures.

**Results:**

This paper describes the isolation of a chitosanase-producing, cold-active bacterium affiliated to the genus *Janthinobacterium*. The 876 bp chitosanase gene from the *Janthinobacterium *strain was isolated and characterized. The chitosanase was related to the Glycosyl Hydrolase family 46 chitosanases with *Streptomyces *chitosanase as the closest related (64% amino acid sequence identity). The chitosanase was expressed recombinantly as a periplasmic enzyme in *Escherichia coli *in amounts about 500 fold greater than in the native *Janthinobacterium *strain. Determination of temperature and pH optimum showed that the native and the recombinant chitosanase have maximal activity at pH 5-7 and at 45°C, but with 30-70% of the maximum activity at 10°C and 30°C, respectively.

**Conclusions:**

A novel chitosanase enzyme and its corresponding gene was isolated from *Janthinobacterium *and produced recombinantly in *E. coli *as a periplasmic enzyme. The *Janthinobacterium *chitosanase displayed reasonable activity at 10°C to 30°C, temperatures that are preferred in transfection and transformation experiments.

## Background

Chitin, a polymer of acetylated β-(1,4)-linked glucosamine (GlcNAc) residues, is the second-most abundant polysaccharide in nature, where it constitutes the major structural component in a number of organisms, *e.g*. crustaceans, insects, nematodes and fungi. Chitosan, which is a partly deacetylated form of chitin, is less abundant but may be found in the cell wall of certain fungi, *e.g. Zygomycetes *[1,2] and green algae like *Chlorella *[3]. Chitin and chitosan have similar molecular structures, since both polysaccharides are made up of β-(1,4)-linked glucosamine (GlcN) residues, which are 50-100% acetylated (chitin) or 0-50% acetylated (chitosan) [4]. In recent years, interest in oligosaccharides derived from chitin and especially from chitosan has increased considerably because these oligosaccharides are water-soluble and possess useful biological activities like antitumor and antimicrobial activities [5-10]. The oligosaccharides may be produced by chemical treatment of polymeric chitosan or may be derived from enzymatic hydrolysis of chitosan.

Enzymes capable of hydrolyzing chitosan, chitosanases (EC 3.2.1.132), may be found in a number of organisms, particularly in microorganisms. Chitosanases are classified into five glycoside hydrolase families: GH-5, GH-8, GH-46, GH-75 and GH-80 [11]. Enzymes from families GH-5 and GH-8 may hydrolyze a number of other glycosides besides chitosan, whereas glycoside hydrolase families GH-46, GH-75 and GH-80 only comprise chitosanases. Among these true chitinases, the chitosanases from *Streptomyces *and *Bacillus *have been studied in detail with respect to *e.g*. catalytic features and molecular structure [12-15]. In addition to members of the genera *Streptomyces *and *Bacillus*, chitosanases have been isolated from a number of other Gram-positive and Gram-negative bacteria *e.g. Acinetobacter *sp. [16], *Amycolatopsis *sp. [17], *Serratia marcescens *[18], *Pseudomonas *sp. [19], *Nocardioides *sp. [20], *Microbacterium *sp. [21], as well as from fungi, *e.g. Gongronella *sp. [22], *Aspergillus oryzae *[23] and *Fusarium solani *[24].

Normally, gene delivery using chitosan microparticles will result in low transfection frequency compared to liposome mediated gene delivery [25]. However, recently it has been shown that gene delivery of DNA complexed with chitosan into mammalian cells may be enhanced if a fungal chitosanase gene is co-expressed inside the cells [26]. Similarly, hydrolytic enzymes, *e.g*. chitosanases, are used for transformation of filamentous fungi by modification of the cell wall structure, making the cell more accessible. So far, the chitosanases, which have been used in such transformation and transfection experiments, have shown optimal temperatures around 60°C, and the enzymes are hardly active at the low temperatures, which are preferable in transfection and transformation experiments. Therefore, we have screened a bacterial strain collection from Greenland and isolated a chitosanase-producing, cold-active *Janthinobacterium *sp. isolate. In this paper, we describe the isolation and characterization of a new chitosanase and we show that the enzyme may be produced heterologously in *Escherichia coli *resulting in high yields.

## Results

### Isolation of chitosanase enzyme and gene from *Janthinobacterium *sp

**Figure 1:**
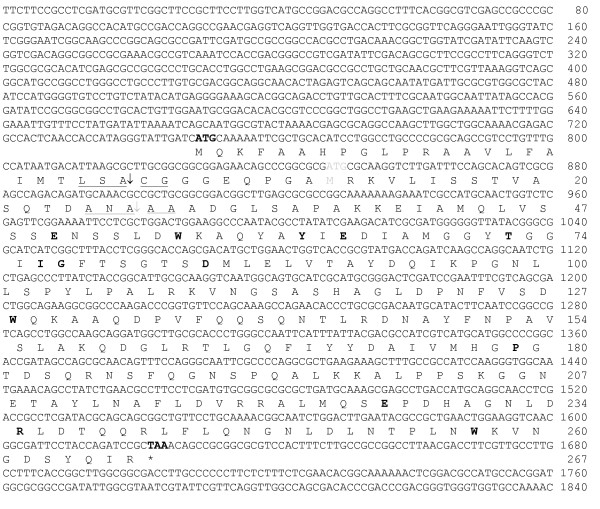
**DNA sequence of chromosomal region encoding chitosanase Cho4239-1 and deduced amino acid sequence**. Nucleotide numbers and amino acid numbers are indicated, omitting the first 24 amino acid residues suggested to function as a signal peptide. The translation initiation codon (ATG) and the stop codon (TAA) of the open reading frame are shown in bold. A predicted signal peptidase II recognition sequence is underlined, and a vertical arrow indicates the putative signal peptide cleavage site. An alternative ATG initiation codon and the corresponding signal peptidase I recognition sequence and cleavage site is underlined with gray. Amino acid residues believed to have significant function in substrate binding, catalysis and structural conformation are shown in bold. Substrate binding subsites B (Pro_179_), C (Gly_77_;Asp_84_), D (Ile_76_;Glu_226_) and E (Tyr_62_) are conserved among chitosanases with residues in subsites C and E as characteristics of glycosyl hydrolase family 46. Also the catalytic glutamic acid (Glu_50_) is conserved but the corresponding aspartic acid which is conserved among other chitosanases could not be identified here. The alternative active site residues (Glu_64 _and Thr_72_; [29]) are indicated in bold. Protein stabilizing residues identified in other chitosanases were also found to be conserved here (Trp_56_;Trp_128_;Arg_235_;Trp_257_) [12,13].

Three thousand bacterial isolates were screened for chitosanase activity on agar plates with chitosan. Thirty isolates were found to produce clear haloes around the colonies after four days of incubation at 20°C. Phylogenetic analysis showed that the isolates, which produced the largest haloes were related to *Janthinobacterium*, *Pedobacter *and *Eubacterium*. One isolate, no. 4239, from a fresh water lake close to Kangerlussuaq in West Greenland produced the largest clearing haloes and was selected for further investigations. Phylogenetic analysis by 16S rRNA sequencing showed that the isolate was affiliated to the genus *Janthinobacterium*. A gene library of *Janthinobacterium *sp. 4239 was constructed in pUC18 in *E. coli *and plated onto agar medium including chitosan. After four days of incubation at 20°C, two transformants, which produced small haloes, were identified. Restriction enzyme analysis indicated that the plasmids in the two clones were identical, and one of the plasmids, pMGJ1061, was selected for subsequent sequence analysis and subcloning. Sequencing of the insert revealed two open reading frames, one encoding the chitosanase (Fig. 1) and one encoding a putative aspartate decarboxylase with reverse orientation (not shown). The open reading frame coding for a chitosanase contained two ATG codons that may both work as translation start codons. Initiation at the first ATG codon (bp 749-751) will result in a polypeptide of 291 amino acid residues (876 bp) including a signal peptide of 24 amino acid residues as determined by SignalP and LipoP algorithms. The amino acid residues around the proposed cleavage site showed similarity to lipoprotein like sites for signal peptidase II cleavage (Fig. 1). Initiation at the second ATG codon (bp 848-850) results in a polypeptide of 258 amino acid residues comprising a putative signal peptide of 18 amino acid residues with a lipoprotein like site for signal peptidase I cleavage (Fig. 1). BLASTP analysis of the mature 267 amino acid polypeptide starting at the first initiation codon showed similarity to glycosyl hydrolase family 46 chitosanase sequences (*Streptomyces *sp. 63% [GenBank:P33665] and 64% [GenBank:ZP_05001348]; *Streptosporangium roseum *64% [GenBank:ZP_04476321]; *Amycolatopsis *sp. 64% [GenBank:BAA94840]). Detailed studies of the *Streptomyces *chitosanase protein structure have emphasized the importance of certain amino acid residues in relation to catalysis and structure. The equivalent amino acid residues of the *Janthinobacterium *sp. 4236 chitosanase are shown in bold in the protein sequence presented in Fig. 1.

### Heterologous expression of *Janthinobacterium *chitosanase in *E. coli*

**Figure 2:**
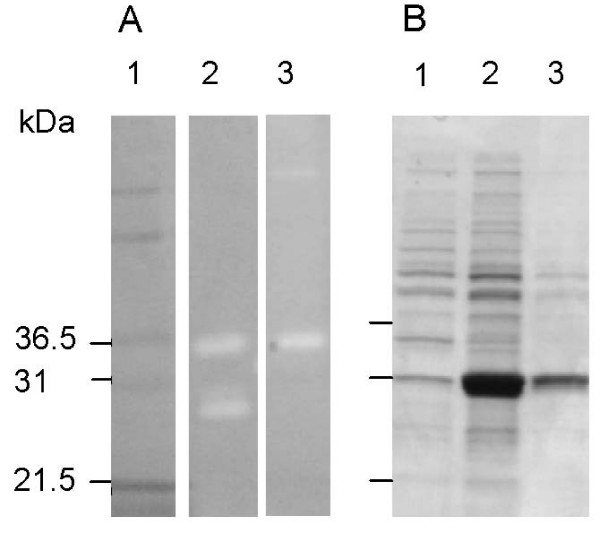
**Zymographic and SDS-PAGE analyses**. (A) Zymogram. Lane 1, molecular weight markers (Invitrogen Mark12). Lane 2, medium from cultivation of *Janthinobacterium *sp. 4239. Lane 3, periplasmic extract of *E. coli *BMS172 diluted 5,000 times. (B) Ordinary SDS polyacrylamide gel. Lane 1, periplasmic extract of reference *E. coli *strain MGJ1042 diluted 5 times. Lane 2, periplasmic extract of reference *E. coli *BMS172 diluted 5 times. Lane 3, same periplasmic extract as in lane 3 diluted 50 times. Notice the difference in migration positions of the *E. coli *BMS172 chitosanase in the two gel systems A and B.

The part of the gene encoding the mature protein (267 amino acids), initiated at the first start codon but without the signal sequence, was further subcloned into an expression vector. In this plasmid, named pBMS172, the chitosanase gene was fused with *E. coli ompA *sequence, substituting the native *Janthiobacterium *signal sequence. Transformants with plasmid pBMS172 showed haloes around the colonies, confirming that the fragment encoded a polypeptide with chitosanase activity. Denaturing zymographic gel electrophoresis and activity staining revealed that the mature secreted product found in the periplasm of BMS172 migrated to a position corresponding to that of the native chitosanase product of 33 kDa (Fig. 2A), isolated from growth medium. In a similar gel system not including the chitosan substrate the same recombinant chitosanse migrated to a position corresponding to 31 kDa, which is in closer agreement with the size of 29 kDa deduced from the sequence analyses (Fig. 2B). The sub-cellular distribution of the chitosanase activity was investigated by determining the enzyme activity in fractionated cellular compartments. Recombinant *E. coli *harbouring plasmid pBMS172 expressed chitosanase activity in about 500 fold greater amounts per cell when compared to the native *Janthinobacterium *sp. 4239 isolate (Table 1). From 50 ml of flask culture a raw periplasmic extract of 1.25 ml was obtained. This extract contained 6.0-6.5 mg recombinant chitosanase protein and provided 3.0-3.5 mg highly purified chitosanase product. Over-expression of chitosanase and periplasmic accumulation of the enzyme posed considerable stress on the *E. coli *transformant. This effect was evident from the high ratio observed between periplasmic and cytoplasmic activity of glucose-6-phosphate dehydrogenase (G6PD, a cytoplasmic marker), when compared to a control strain (Table 1). Preliminary characterization experiments with the recombinant chitosanase produced in *E. coli *BMS172 gave results similar to those obtained with the native 33 kDa enzyme (data not shown). Therefore, since purification of periplasmic chitosanase was easier, detailed characterization of enzyme performance was carried out on chitosanase from *E. coli *pBMS172.

**Table 1:** Cellular distribution of chitosanase activity

	Chitosanase specific activity^a ^units·OD_600_^-1^·ml^-1^			G6PD activity^b ^%
**Strains**	CYT	PER	MED	PER/CYT

*Janthinobacterium *4239	0.016	0.007	0.035	-

*E. coli *BMS172	17.8	20.3	1.25	62

*E. coli *MGJ1042 (control)	0.002	0	0	23

^a ^Specific chitosanase activity is presented as units (μmol/min) of reducing ends released per milliliter of cells with OD_600 _= 1 (U·OD_600_^-1^·ml^-1^). CYT, cytoplasmic extract. PER, periplasmic extract. MED, culture medium obtained after centrifugation. ^b ^Glucose-6-phosphate dehydrogenase (G6PD) is a cytoplasmic marker indicating the degree of cell lysis. Periplasmic extract of *Janthinobacterium *sp. strain 4239 showed no G6PD activity.

### Characterization of chitosanase from *Janthinobacterium*

**Figure 3:**
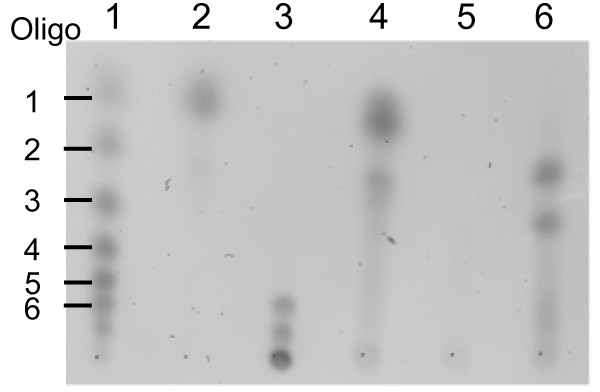
**TLC analysis of products from hydrolysis of a chitosan hexamer**. Lane 1, series of purified chitosan-oligosaccharides from monomer to hexamer (indicated to the right of zymogram). Lane 2, glucosamine monomer. Lane 3, chitosan hexamer incubated with an extracellular extract from *E. coli *(MGJ1042). Lane 4, chitosan hexamer incubated with an extracellular extract from native *Janthinobacterium *sp. 4239. Lane 5, extracellular extract from *E. coli *(MGJ1042). Lane 6, chitosan hexamer incubated with an extracellular extract from recombinant *E. coli *MGJ1061.

Purified chitosanase produced by *E. coli *pBMS172 was analyzed with respect to substrate specificity. The recombinant chitosanase displayed high relative activity on chitosan with 80% deacetylation and a peak molecular weight of 1000 kDa (Table 2). The specific activity on this substrate was found to be 1500 U/mg (45°C). Slightly lower activity was observed on chitosan with lower peak molecular weight (500 kDa and 700 kDa) and on 55% deacetylated chitosan. No activity was observed on chitin, cellulose, glucan, xylan or arabinoxylan substrates (Table 2). TLC analysis of the products from hydrolysis of chitosan hexamer showed that digestion with recombinant chitosanase resulted in only dimers and trimers of glucosamine. In contrast, concentrated medium from *Janthinobacterium *sp. 4239 culture was able to hydrolyze the hexameric substrate into monomers and dimers (Fig. 3).

**Table 2:** Relative chitosanase activity on different substrates

Substrate	Relative act (%)
Chitosan 80% DD, 1000 kDa	100

Chitosan 80% DD, 700 kDa	87

Chitosan 80% DD, 500 kDa	84

Chitosan 55% DD	68

Colloidal chitin	0

Insoluble cellulose	0

CM cellulose	0

Beta-1,3-glucan	0

Xylan	0

Arabinoxylan	0

DD, degree of deacetylation. CM, carboxymethylated

**Figure 4:**
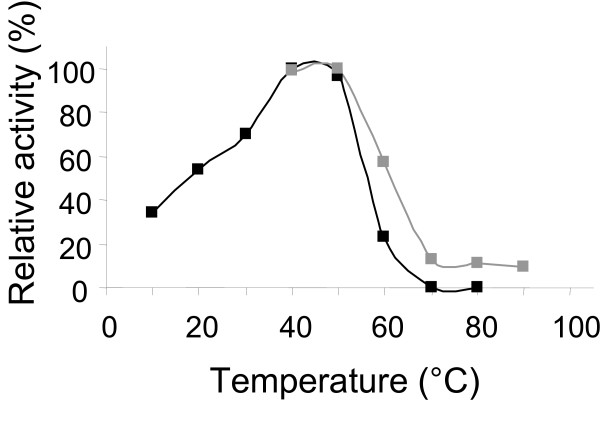
**Influence of temperature on chitosanase activity and stability**. Black squares and lines: Relative activity. Gray squares and lines: relative activity after incubation at the same temperature for 30 min.

**Figure 5:**
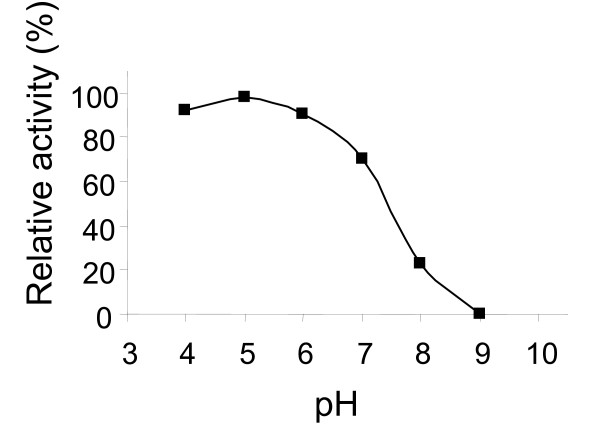
**Influence of pH on chitosanase activity**. Relative activity was determined using 80% deacetylatd chitosan.

Determination of temperature optimum was conducted with 80% deacetylated chitosan substrate (Fig.4). Purified chitosanase from *E. coli *pBMS172 displayed maximal activity at 45°C and 30-70% of the maximal activity was observed at 10-30°C. The recombinant chitosanase was stable at temperatures up to 50°C above which the enzyme activity decreased rapidly and hardly remained after treatment at 70°C for 30 min. Determination of the pH optimum was conducted with 80% deacetylated chitosan. The chitosanase displayed a broad pH optimum between pH 5 and 7 (Fig. 5). Hardly any activity was left at pH 8 and above.

## Discussion

Previously, *Janthinobacterium *strains degrading chitin have been reported [27,28] but this report is the first to describe a chitosanase from the genus *Janthinobacterium*. Polyacrylamide gel electrophoresis and zymographic staining revealed that two polypeptides with the apparent molecular weights of 27.5 and 33 kDa were able to hydrolyze chitosan (Fig. 2A, lane 2). This observation suggested that *Janthinobacterium *sp. strain 4239 harbours two different chitosanase genes, and that in the subsequent screening of the genomic library in *E. coli *only one of the genes were isolated in this study. However, another explanation for the two bands in extracellular extracts from native *Janthinobacterium *sp. 4239 cells may be that the strain produces two enzyme variants from the isolated *cho4239-1 *gene by using two different translation initiation positions and/or by cleavage at two different positions in the polypeptide chain. Apparently, the presence of chitosan substrate in the zymographic gel influences the migration position of the recombinant chitosanase product. The 27.5 kDa chitosan hydrolytic product from the native *Janthinobacterium *sp. 4239 may also not be size estimated entirely correctly in the zymogram. If this second chitosan hydrolyzing protein is a result of translational initiation at the second putative start position the expected molecular size is expected to be 28.1 kDa. However, we can not from our data conclude if *Janthinobacterium *sp. 4239 harbours one or two chitosanase gene(s).

Expression of the *Janthinobacterium *chitosanase in *E. coli *resulted in large amounts of recombinant chitosanase in the periplasm (BMS172). Also, a large amount of recombinant protein accumulated in the cytoplasm, suggesting that the efficiency of the expression system could be further improved by introducing elements that promote secretion. SDS-PAGE showed that the monomeric molecular weight of the chitosanase secreted by the BMS172 construct was 33 kDa, which is similarly to the larger of the two products secreted by the native *Janthinobacterium *sp. strain 4239 (Fig. 2, lane 2).

In agreement with the affiliation to glycosyl hydrolase family 46, based on protein homology, the ability of the chitosanase to convert chitosan hexamers into dimers and trimers further emphasizes the relation with endo-chitosanases. However, hexamer substrate conversion by crude extracellular protein from *Janthinobacterium *sp. 4239 resulted in monomeric glucosamine accumulation, suggesting that the native bacterium also produces exo-beta-D-glucosaminidase activity in addition to endo-hydrolytic chitosanase activity.

The chitosanase displayed 64% amino acid sequence identity with the well-characterized chitosanase from *Streptomyces *sp. N174. Alignment of the *Janthinobacterium *sp. 4239 chitosanase with that of *Streptomyces *sp. N174 revealed the presence of a putative catalytic residue at Glu_22 _(*Streptomyces *numbering). However, an equivalent of Asp_40_, which is also believed to be involved in catalysis in most - if not all - chitosanases from family GH-46, was not found. However, it has been shown [29] that an Asp to Gly mutation in position 40 in *Streptomyces *sp. N174 chitosanase did not affect the stereochemical mechanism of catalysis or the mode of interaction with the substrate. In stead, other accessory active site residues, Glu_36 _and Thr_45_, were shown to be involved in catalysis. Examination of the *Janthinobacterium *sp. 4239 chitosanase sequence (Fig. 1) shows the presence of a glutamine residue similar to the Glu_36 _and a threonine one residue from the Thr_45 _residue in the *Streptomyces *sp. N174 chitosanase. Thus, it may be possible that the *Janthinobacterium *sp. 4239 chitosanase uses the Glu_36 _residue as a catalytic residue.

## Conclusions

This study provides a chitosanase affiliated to glycosyl hydrolase family 46 with specificity towards chitosan substrate and with activity at conditions favourable for transformation and transfection experiments. The ease, at which the chitosanase can be produced in *E. coli *and accumulated in the periplasm, provides a product with few impurities suitable for many experimental procedures. The application value of this enzyme can now be tested in protocols relying on efficient hydrolysis at low to moderate temperatures.

## Methods

### Materials

Chitosan samples with peak molecular weights of 500,000, 700,000 and 1,000,000 g/mol, respectively, and all with a degree of deacetylation of more than 80% were obtained from Cognis Deutschland GmbH&Co., KG (Düsseldorf, Germany). The chitosan (more than 80% deacetylated) used for preparing screening plates was obtained from Carbomer (Westborough, MA, USA). A specified preparation of water-soluble chitosan (WSC) with peak molecular weight of 2,500,000 g/mol and a degree of deacetylation of 55% was obtained from Primex ehf. (Reykjavik, Iceland). Carboxymethylcellulose and Avicel were obtained from Sigma-Aldrich. Crab flakes for colloidal chitin preparation were obtained from Carbomer (Westborough, MA, USA). The chitosan-oligosaccharides were obtained from Seikagaku Corporation (Japan). Restriction enzymes were from New England Biolabs and Fermentas.

### Isolation and characterization of chitosanase-producing strain

Samples of microorganisms were collected at various locations along the West-coast of Greenland in 2000. Soil, sediment and water samples were plated on M9 medium supplemented with different carbohydrates. Strain 4239 was isolated from a fresh water lake close to Kangerlussuaq. The screening medium was prepared from stocks. A chitosan stock for one liter of medium was obtained by autoclaving 1.8 g of insoluble chitosan in 200 ml of demineralized water. Once room temperature was reached, the chitosan was brought in solution by adding 18 ml of sterile 1 M HCl. The chitosan was dissolved during stirring for two hours and then the entire stock was poured into 700 ml of autoclaved warm, sterile medium at a slow rate during vigorous stirring in order to maintain small colloidal particles. To prepare 700 ml of medium, 9 g of KH_2_PO_4_, 6 g of K_2_HPO_4_, 8 g of tryptone (Difco), 4 g of yeast extract (Difco) and 15 g of agar was mixed. The medium was further supplemented with 2 ml of Vogels Trace Elements (5 g of citric acid·H_2_O, 5 g of ZnSO_4_·7H_2_O, 1 g of Fe(NH_4_)_2_(SO_4_)_2_·6H_2_O, 0.25 g of CuSO_4_·5H_2_O, 0.05 g of MnSO_4_·H_2_O, 0.05 g of H_3_BO_4 _and 0.05 g of Na_2_MoO_4_·2H_2_O per 100 ml, sterilized by filtration). Finally, 2 ml of a sterile solution of MgSO_4_·6H_2_O (203 g/l) was added. Before use, the final volume was adjusted to 1 liter with sterile water. The isolated *Janthinobacterium *sp. strain 4239 was propagated for four days at 20°C in baffled, conical culture flasks containing the complete chitosan growth medium without agar and aerated by shaking. Supernatants were concentrated on spin filters (Amicon, 10 kDa cut-off) and analyzed for protein content and activity. Material for DNA isolation was obtained by incubation of cells for two days in a medium, in which the chitosan was substituted by 2.5 ml of 20% glucose per liter. Screening of *E. coli *transformants expressing chitosanase was carried out on LB agar plates supplemented with 1.8 g/l of chitosan prepared as described above.

### Phylogenetic analysis

The 16S PCR product was generated with the primers presented in Table 3. The product was sequenced by primer walking and the information was deposited in GenBank [GenBank:GQ487532].

**Table 3:** Bacteria, plasmids and oligonucleotide primers

Name	Characteristics	Reference or origin
***Janthinobacterium *sp. 4239**		

	Gram negative, chitosanolytic, no chitinolytic activity detected.	This study

***E.coli***		

DH10B	F' *mcr*A Δ(*mrr*-*hsd*RMS-*mcr*BC) Φ80*lac*ZΔM15 Δ*lac*X74 *deo*R *rec*A1 *end*A1 *ara*D139 Δ(*ara, leu*)7697 *gal*U *gal*K λ^- ^*rps*L (Streptomycin R) *nup*G	Invitrogen

MGJ1042	DH10B:pMGJ1042. Reference strain.	This study

MGJ1061	DH10B:pMGJ1061. Chromosomal library clone expressing Cho4239-1.	This study

BMS172	DH10B:pBMS172. Construction expressing Cho4239-1 fused with the OmpA signal peptide.	This study

**Plasmids**		

pUC18	*lac*UV5 promoter, Amp^r^, ColEI origin, 2.69 kb.	[40]

pMGJ1042	pMF1::OmpA-*Sph*I fragment. *tet*^p/o ^promoter, Cm^r^, *pro*AB complementation and ColEI replication origin.	[36]

pMGJ1061	pUC18::4 kb chromosomal fragment encoding Cho4239-1.	This study

pBMS172	pMGJ1042::815 bp PCR fragment digested with *Sph*I/*Bam*HI and fused with OmpA signal peptide.	This study

**Oligonucleotide primers**		

chodel4239-1.for	5'-ACATGCATGCGGCGGCGGAGAACAGC-3'	Construction of pBMS172

cho4239-1.rev3	5'-CGCGGATCCGCGGCTGTTTAGCGGATCTGGT-3'	Construction of pBMS172

616V	5'-AGAGTTTGATYMTGGCTCAG-3'	16S RNA sequence

630R	5'-CAKAAAGGAGGTGATCC-3'	16S RNA sequence

### Molecular cloning of chitosanase gene

Isolation of chromosomal DNA from *Janthinobacterium *sp. 4239 was carried out from a 100 ml cell culture which was harvested at OD_600 _= 3. The cells were harvested by centrifugation, resuspended in 10 ml of TE buffer (10 mM Tris·HCl, pH 7.5, 1 mM EDTA) and frozen at -20°C. The cells were thawed and incubated with 1% (w/v) SDS, 2 mg of proteinase K (Roche) and 0.1 mg of RNaseA. After incubation at room temperature for 30 min, 1.8 ml of 5 M NaCl was added. Phase separation was enhanced by the addition of 1.5 ml of 10% (w/v) hexadecyl-trimethyl ammonium bromide (CTAB) dissolved in 0.7 M NaCl. The subsequent extraction steps were conducted essentially as described by Marmur [30]. Chromosomal DNA was isolated after two rounds of phenol/chloroform/isoamyl alcohol extractions and two chloroform/isoamyl alcohol extractions, followed by addition of one volume of 2-propanol for precipitation. Finally, the DNA was spooled onto a sterile inoculation needle and washed several times in 70% (v/v) ethanol. The yield of purified DNA obtained from a 100 ml cell culture was estimated to 200 μg. The DNA was dissolved in TE-buffer and stored at 5°C.

A DNA library was constructed by partial digestion of 24 μg of chromosomal DNA using 0.8 units of the restriction enzyme *Bsp*1431 (an isoscizomer of *Sau*3A). After 15 min the partially digested DNA was heat treated at 65°C for 10 min. DNA fragments above 3 kb in size were purified by agarose gel electrophoresis. The fragments were ligated into plasmid pUC18 digested with the restriction enzyme *Bam*HI, and the ligation mixture was transformed into electrocompetent *E. coli *strain DH10B cells (Gibco BRL). A total of 40,000 transformants were plated onto LB plates supplemented with 1.8 g of chitosan per liter. *E. coli *colonies expressing chitosanase were identified by the appearance of clearing zones around the colonies.

### Nucleotide sequence analysis

One of the *E. coli *isolates contained a pUC18 plasmid with a 4 kb insert, plasmid pMGJ1061. DNA sequencing of the insert in pMGJ1061 was carried out using custom ordered primers from DNA Technology (Aarhus, Denmark). Reactions were analyzed with an ALF Express sequencer (Pharmacia). Inserts were sequenced on both strands using plasmid specific primers and primer walking. Basic DNA analysis was carried out in DNA Star (Lasergene) and database searches for homologous sequences were carried out using the BLAST program [31]. Multiple alignment of amino acid sequences was performed with the ClustalX program [32]. A putative N-terminal signal peptide was identified by SignalP version 3.0 and LipoP server facilities [33]. The DNA sequence of *Janthinobacterium *sp. 4239 chitosanase, Cho4239-1, was deposited in GenBank [GenBank:GQ487533].

### Subcloning and expression in *E. coli*

Heterologous expression of *Janthinobacterium *sp. 4239 chitosanase in *E. coli *was carried out using vector pMGJ1042, a derivative of plasmid pMF1 [34,35]. Secretion from this plasmid has been improved by introduction of the native OmpA signal peptide cleavage site as described previously [36]. The chitosanase gene from *Janthinobacterium *sp. 4239 was isolated by PCR with primers specific for the *cho4239-1 *gene (Table 3). The DNA fragment was restricted with *Sph*I and *Bam*HI and inserted into pMGJ1042 also digested with these enzymes. In this plasmid, pBMS172, the putative catalytic part of the chitosanase gene was fused to the OmpA. The insert of plasmid pBMS172 was sequenced, and growth experiments on chitosan-containing agar plates with chloramphenicol (30 μg/ml) and tetracycline (10 ng/ml) confirmed the identity of the plasmid (Table 3).

Recombinant chitosanase was produced from 50 ml cultures of *E. coli *cells harbouring plasmid pBMS172. The cells were grown in enforced medium (16 g bactotryptone (Difco), 16 g yeast extract (Difco), 5 g NaCl, 2.5 g K_2_HPO_4 _per liter) and when the OD_600 _reached 2, the cultures were induced with 250 μg/ml of anhydro-tetracycline for 5-5.5 h.

### Assay procedures

Chitosanase activity was determined as the rate at which reducing molecules were generated. Reducing ends were quantitated by reaction with potassium ferricyanide stain [37] and converted to molecules by reference to a standard curve of reduced stain intensity versus different amounts of GlcN. The buffer systems outlined by Keith and Morrison [38] were used for testing activity at different pH values. The highly deacetylated chitosan substrates were dissolved in 25 mM HCl before adjusting the assay pH. In standard assay conditions substrate chitosan with a peak molecular weight of 1,000 kDa and 80% deacetylation was dissolved in HCl and equilibrated to pH 5.5 with 150 mM NH_4_Ac-buffer. One unit of chitosanase was defined as the amount of enzyme that liberates 1 μmol of d-glucosamine per min under the conditions described above. The glucose-6-phosphate dehydrogenase (G6PD) activity was assayed in extracts from an equal number of cells. In this assay the formation of NADH during glucose-6-phophate conversion was determined at 340 nm.

### Extraction and purification of *Janthinobacterium *chitosanase

Bacterial cell pellets were obtained by centrifugation and then mixed with an equal volume of extraction buffer (2.5 mM EDTA and 20 mM Tris·HCl, pH 6.5). Three successive cycles of freezing and thawing were conducted for the specific extraction of periplasmatic proteins [39]. After precipitation of the remaining cell content, the cytoplasm was extracted in the same buffer by FastPrep (Bio101) treatment including a 1:1 volume ratio of 102 nm glass beads (Sigma) at speed 5.5 for three times 20 sec. For long-term storage, the extracts were preserved with 50% (v/v) glycerol and kept at -20°C. For storage less than a week, the extract were preserved by addition of 1.5 mM NaN_3 _and kept in the refrigerator. Chitosanase was purified from periplasmic extract by gel filtration on a Superdex 75 HR 10/30 column (GE Healthcare). The column was equilibrated and eluted with 50 mM potassium phosphate, 150 mM NaCl, pH 6.5. Chitosanase containing fractions were pooled and analyzed by SDS-PAGE (Invitrogen). The quantity of the purified chitosanase protein was estimated relative to the intensity of molecular weight markers (Mark12, Invitrogen).

### Characterization of chitosanase

Zymographic polyacrylamide gels were cast with 30% ProtoGel 37.5:1 and ProtoGel buffers from National Diagnostics. In the separation gel 0.025% (w/v) WSC chitosan was included. Prior to loading the gels, the enzyme samples were denatured by heat treatment for 3 min at 100°C. The sample buffer was prepared as a two-fold stock solution by mixing 15 ml of 50% (w/v) sucrose, 10 ml of 10% (w/v) SDS and 5 ml of 1 M Tris·HCl, pH 6.8. After electrophoresis, gels were renatured by overnight incubation in phosphate buffer, pH 6.5, stained with 0.1% Fluorescent Brightner 28 (Sigma) in 0.5 M Tris·HCl, pH 9 for 5 min, and destained in several changes of demineralized water for a period of 3-4 h. Zymographic gel section with marker proteins and acrylamide gels without chitosan substrate were stained with Coomassie Brilliant Blue. Results were documented by UV (312 nm) exposure and photography. TLC experiments were conducted on Silica Gel 60 plates (Merck) with oligosaccharides and glucosamine as references. The mobile phase was prepared by mixing 100 ml of 1-propanol and 50 ml of 25% (v/v) ammonia (Merck). The separation was conducted over two runs each taking about 6 h. After drying, the plates were sprayed with 5% (v/v) HCl in ethanol and heated for 10 min in an oven at 170°C.

## Competing interests

The authors declare that they have no competing interests.

## Authors' contributions

PS coordinated the project, planned the field trip and sampled the biological material. PS and MGJ established the strain collection. MGJ screened the strain collection, isolated chitosanase-producing bacteria and performed all molecular biology work. OCH conducted all protein purifications. All authors have read and approved the final version of the manuscript.
